# The Essential *yhcSR* Two-Component Signal Transduction System Directly Regulates the *lac* and *opuCABCD* Operons of *Staphylococcus aureus*

**DOI:** 10.1371/journal.pone.0050608

**Published:** 2012-11-30

**Authors:** Meiying Yan, Jeffrey W. Hall, Junshu Yang, Yinduo Ji

**Affiliations:** Department of Veterinary and Biomedical Sciences, College of Veterinary Medicine, University of Minnesota, St. Paul, Minnesota, United States of America; National Institutes of Health, United States of America

## Abstract

Our previous studies suggested that the essential two-component signal transduction system, YhcSR, regulates the *opuCABCD* operon at the transcriptional level, and the *Pspac*-driven *opuCABCD* partially complements the lethal effects of *yhcS* antisense RNA expression in *Staphylococcus aureus*. However, the reason why *yhcSR* regulon is required for growth is still unclear. In this report, we present that the *lac* and *opuC* operons are directly transcriptionally regulated by YhcSR. Using real-time RT-PCR we showed that the down-regulation of *yhcSR* expression affected the transcription of *lacA* encoding galactose-6-phosphotase isomerase subunit LacA, and *opuCA* encoding a subunit of a glycine betaine/carnitine/choline ABC transporter. Promoter-*lux* reporter fusion studies further confirmed the transcriptional regulation of *lac* by YhcSR. Gel shift assays revealed that YhcR binds to the promoter regions of the *lac* and *opuC* operons. Moreover, the *Pspac*-driven *lacABC* expression *in trans* was able to partially complement the lethal effect of induced *yhcS* antisense RNA. Likewise, the *Pspac*-driven *opuCABCD* expression *in trans* complemented the growth defect of *S. aureus* in a high osmotic strength medium during the depletion of YhcSR. Taken together, the above data indicate that the *yhcSR* system directly regulates the expression of *lac* and *opuC* operons, which, in turn, may be partially associated with the essentiality of *yhcSR* in *S. aureus*. These results provide a new insight into the biological functions of the *yhcSR*, a global regulator.

## Introduction

The continuing increase of hospital- and community-associated methicillin resistant *Staphylococcus aureus* infections highlights an urgent need for the development of alternative potent antibacterial agents [1]–[3]. The ability of this organism to resist current antibiotic therapies and cause infection is partially due to the coordinated regulation of gene expression allowing the bacteria to survive in different stress conditions. Two-component signal (TCS) transduction systems are important sensory units and allow microbial organisms to adapt to different niches, as well as play a significant role in pathogenesis and biofilm formation for various bacterial species [4]–[7]. Therefore, interrupting these critical signaling pathways may provide an alternative strategy for the development of novel classes of preventive and/or therapeutic antibacterial agents [8].

A typical two-component system is composed of a histidine kinase sensor and a cognate response regulator responsible for sensing and responding to environmental signals by regulating gene expression, respectively [5]. In *S. aureus*, at least 16 different two-component signal systems have been identified [6], and two TCSs, *yycFG* and *yhcSR* (also known as *airSR*), are required for survival [9]–[11]. The *yycFG* system, which has orthologs in *Bacillus subtilis* [12] and *Streptococcus pneumoniae* [13], is the first reported TCS regulatory system that is indispensable for cell viability in *S. aureus* [9]. Analyses of conditional lethal *yycFG* mutants in *B. subtilis* suggested that this system controls the *ftsAZ* operon that is involved in the process of cell-wall division [14], as well as endopeptidase-type autolysins including YvcE and LytE [15]. However, YycFG is not involved in the regulation of *ftsZ* expression in *S. pneumoniae* and *S. aureus*, but, modulates the expression of genes involved in cell wall metabolism in *S. aureus* [16], and an essential gene, *pcsB*, encoding a cysteine, histidine-dependent amidohydrolase/peptidase involved in cell wall biosynthesis in *S. pneumoniae* and *S. aureus* [16]–[20].

We have identified another TCS, *yhcSR*, which is also required for survival of *S. aureus* [10]. However, the reason why *yhcSR* is required for growth is totally unknown. In order to elucidate the biological functions of *yhcSR*, we examined the effect of *yhcSR* on gene expression using a microarray assay. Our preliminary microarray analysis suggested that the down-regulation of *yhcSR* expression affects the expression of genes associated with a variety of biological functions. Recently, we demonstrated that YhcSR positively regulates the *nreABC* and *narGHIJ* operons which are responsible for dissimilatory nitrate reduction under anaerobic growth conditions [21]. We have also previously published data indicating regulation of the *opuCABCD* operon by YhcSR and showed that plasmid-borne expression of OpuCABCD partially complements the lethal effect of induced *yhcS* antisense RNA [22].

In this study, we employed quantitative RT-PCR, promoter-*lux* reporter fusions, and gel-shift technologies and demonstrated that the *yhcSR* system directly regulates the transcription of the *lac* operon encoding the structural genes for lactose and galactose metabolism, and *opuCABCD* operon encoding a glycine betaine/carnitine/choline ABC transporter [23], [24]. Moreover, we found that that the regulation of the *lac* and *opuC* operons may partially contribute to the essentiality of the *yhcSR* regulon in *S. aureus*.

## Materials and Methods

### Bacterial Strains, Plasmids and Growth Media

The bacterial strains and plasmids used in this study are listed in Table 1. The *S. aureus* cells were cultured in Trypticase soy broth (TSB) at 37°C with shaking. *E. coli* strains were grown in Luria-Bertani (LB) medium. Transformants containing recombinant plasmids were selected on LB agar containing ampicillin (100 µg/ml), chloramphenicol (50 µg/ml), or erythromycin (300 µg/ml) for *E. coli*, and TSA containing chloramphenicol (10 µg/ml) or erythromycin (5 µg/ml) for *S. aureus*. The isopropyl-β-D-thiogalactoside (IPTG) was added as 1 mM at final concentration. Where noted, NaCl (1 M) and choline (1 µM) were added to chemically defined medium (CDM).

**Table 1:** Bacterial strains and plasmids used in this study.

Strain or plasmid	Description	Reference or source
*S. aureus* strains		
RN4220	Laboratory strain: *rsbU*	[49]
WCUH29	Clinical human *S. aureus* isolate: *rsbU^+^*	
YJ2002	WCUH29 containing plasmid pYH3	[30]
JAS909	WCUH29 containing plasmid pSAS909 carrying *yhcS* antisense; Erm^r^	[10]
YJ606	WCUH29 containing plasmid pCY606; Erm^r^	[22]
YJ1997	WCUH29 containing plasmid pMY1997; Erm^r^	This study
YJ107	WCUH29 containing plasmid pMY107; Erm^r^	[22]
YJ207	WCUH29 containing plasmid pMY207; Erm^r^	[22]
YJ307	WCUH29 containing plasmid pMY307; Erm^r^	This study
Plasmids		
pET24b	Vector for overproducing His-tagged proteins, Kan^r^	Novagen
pyhcR-24b	pET24b derivative for overproduction of YhcR, Kan^r^	This study
pCY606	Shuttle vector, derived from pSAS909, containing *luxABCDE* and *yhcS* antisense; Erm^r^	[22]
pMY1997	Derived from pCY606, with sa1997 promoter - *luxABCDE* reporter fusion; Erm^r^	This study
pMY107	Derived from pCY606, with *luxABCDE* replaced by *Pspac* promoter region; Erm^r^	[22]
pMY207	Derived from pMY107, with sa2237operon under the *Pspac*; Erm^r^	[22]
pMY307	Derived from pMY107, with sa1997operon under the *Pspac*; Erm^r^	This study

### RNA Isolation and Purification

Overnight cultures of *S. aureus* were inoculated in 1% in TSB medium and grown to the mid-exponential (3 hr) phase of growth. Total RNA was purified from the above culture as described [25]. Briefly, bacterial cells were harvested by centrifugation, and the RNA was isolated by the RNAPrep kit (Promega). Contaminating DNA was removed with a DNA-*free* kit (Ambion), and the RNA yield was determined spectrophotometrically at 260 nm.

### Quantitative Real-time RT-PCR (qPCR) Analysis

In order to determine whether the down-regulation of *yhcSR* expression has any impact on the expression of several identified essential genes, we employed quantitative real-time reverse transcription (RT) PCR to compare the RNA levels, as described [25], [26]. The first strand cDNA was synthesized using reverse transcriptase with the SuperScript III Platinum Two-Step qRT-PCR kit (Invitrogen). For each RNA sample, we performed duplicate reactions of reverse transcription, as well as a control without reverse transcriptase, in order to determine the levels of DNA contamination. PCR reactions were set up in triplicate by using the SYBR Green PCR Master Mix (Stratagene). Real-time sequence-specific detection and relative quantitation were performed with the Stratagene Mx3000P Real Time PCR System. Gene-specific primers were designed to yield ∼100 bp of specific products (Table 2). Relative quantification of the product was calculated using the Comparative C_T_ method, as described for the Stratagene Mx3000P system. The housekeeping gene 16s rRNA was used as an endogenous control [26]. All samples were analyzed in triplicate and normalized against 16s rRNA gene expression. The experiments were repeated at least three times. To determine whether the complementary effect was attributed to the over-expression of a *Pspac*-driven *lacABC* operon, we also utilized the qPCR as the above described.

**Table 2:** Primers used in this study.

Primers	Sequence(5′-3′)
yhcRforNdeI	GGAATTCCATATGAACAAAGTAATATTAGTAG
yhcRrevXhoI	CCGCTCGAGAATCAACTTATTTTCCATTGC
sa1997proNotFor	TAGTGCGGCCGCATTAAAAGTATAACTGCATTG
sa1997proNotRev	TAGTGCGGCCGCTAATAAGACTCCTTTTTACTTT
Sa2237prGSFor416bp	TGCATTATTACAAAAATTCGAC
Sa2237prGSRev416bp	AACATAATCATTTCTCCTTCC
Sa2237prGSFor312bp	TATGAGTTATCTATTTAGTTGC
Sa2237prGSFor210bp	AGTAATCGGTAGAAATTCAAC
Sa2237prGSFor172bp	TACTGTTAAGTATTCACATTAC
SA1997prGSfor	TTAAAAGTATAACTGCATTG
SA1997prGSrev	CATCTGAACCAATAATAATC
Pspacfor	TCTAGAGCTGCCTGCCGCGTTTCGGT
Pspacrev	GCTTGAATTCCCGGGCGGCCGCCGCGGCCGGCCAATTGTTATCCG CTCACAATTC
Sa1997RBSfor	TAGTGCGGCCGCGGCCGGCCAGGAGGGAGTCTTATT ATGGCGATTATTATTGG
Sa1995rev	TAGTGCGGCCGCGGCCGGCC TTACACCTCTAAAACTTCAATTTG
SA2237RTfor	CGTATCGGTGTCGTAAGAGCACT
SA2237RTrev	GCACCACCTTACCTTCTGACAT
SA1997RTfor	TGG TGC AGG TAG CTT TAT GGT TG
SA1997RTrev	GCA TAT CTA CGC GGA TTT GGT GT
Sa0222-for	AAG CAG TTA AAG AAG CAG ACG AAT CTT G
Sa0222-rev	GTT GTG TTG TTT CTT CAG CTT TAC CAG
SA0959RTfor	GGA TAC ACC AGG ACA TGC AGA CTT
SA0959RTrev	CTA CAA CAC CCT CTG GAC GTG CT
SA1044RTfor	CCT AAC ACA AGA CCG GTA CCA GAT
SA1044RTrev	CTT CAC AGT GTG CTA CGA TGG CTT
P2290-F	TGT CGT CTT GAA ATA CGG CTG T
P2290-R	CTA TAT TGT TCG GTT TTT AAA AGC AAT G
SA1269RTfor	GCC GTC AAG AGA GGC ATT TGA AG
SA1269RTrev	CAA GAC CAC CTG CTC CTA CAA CAA
SA1271RTfor	GAT GCA ACG ATT GTG ATG CCA G
SA1271RTfor	ATT AGG CCA CCT CCA CCA ACT G

### Construction of *lac* Promoter*-lux* Reporter Fusion

In order to further confirm whether the *yhcSR* regulatory system transcriptionally regulates the expression of *lac* we created a promoter-*lux* reporter fusion using the *yhcS* antisense expression vector, pCY606 as described and previously used to build an *opuC* promoter reporter [22]. The promoter region of structural *lac* genes was amplified by PCR respectively using the primers (Sa1997proNotFor/Sa1997proNotrev) listed in Table 2, digested with *NotI* and ligated upstream of the promoterless *luxABCDE* of pCY606, which was digested with the same enzymes. The resulting recombinant plasmids pMY1997 containing the promoter-*lux* reporter fusion was transformed into *E. coli* DH10B competent cells and correct orientation and DNA sequence was confirmed by PCR, restriction enzyme digestion, and DNA sequencing. The plasmid pMY1997 was purified and electroporated into *S. aureus* RN4220, and then into WCUH29, resulting in *S. aureus* strain, YJ1997. The *lux* expression was monitored during growth in TSB at 37°C with a Chiron luminometer. The relative light units (RLU) were calculated (bioluminescence intensity/optical density at 600 nm). Each experiment was repeated at least three times.

### Cloning, Expression and Purification of YhcR-His Tagged Fusion Protein

In order to differentiate which identified genes are directly regulated by the *yhcSR* regulatory system, we cloned and purified a His-Tagged YhcR response regulator protein as described. The *yhcR* coding region was obtained by PCR from *S. aureus* and cloned into *Nde*I and *Xho*I sites of the *E. coli* expression vector pET24b. The recombinant plasmid (pET*yhcR*) was confirmed by PCR and DNA sequencing (data not shown) and transformed into *E. coli* strain BL21(DE3). The transformants were incubated until mid-log phase (OD600 nm = ∼0.6) followed by induction of *yhcR* expression by adding IPTG (final concentration 1 mM). After two hours of incubation, cells were harvested and lysed by sonication. The expression of YhcR was confirmed by SDS-PAGE followed by Coomassie Bright Blue staining (data not shown).

To purify the YhcR-His tagged protein a 500 ml culture of the BL21 (DE3) containing pET*yhcR* was induced, and the cell pellet was collected and lysed in Lysis Buffer (8 M Urea, 0.1 M NaH_2_PO_4_, 0.01 M Tris-Cl, pH8.0) by incubation at room temperature for 1 h with agitation. The supernatant was collected by centrifugation, applied to the Ni-NTA His-Binding Resin, and incubated for 30 min with shaking. The resin mixture was loaded onto the column and washed twice with washing buffer (8 M urea, 0.1 M NaH_2_PO_4_, 0.01 M Tris-Cl, pH6.3). The YhcR-His protein was eluted with elution buffer (8 M urea, 0.1 M NaH_2_PO_4_, 0.01 M Tris-Cl, pH5.6). Following Ni-NTA affinity purification the YhcR-His tag protein was further purified by size exclusion chromatography using Sephadex-50 (Sigma). The purified YhcR-His protein was confirmed by SDS-PAGE followed by Coomassie Bright Blue staining (data not shown). The concentration of purified YhcR-His protein was determined by the Bradford method.

### Protein Phosphorylation *in vitro*

For YhcR phosphorylation *in vitro*, total of 3 µg of protein was incubated in 30 µl of phosphorylation buffer [20 mM NaH_2_PO_4_ (pH8.0), 10 mM MgCl_2_, 1 mM DTT, 32 mM acetyl phosphate] at 37°C for 90 min as described [27].

### Gel Mobility Shift DNA-binding Assay

To determine which identified gene(s) are directly regulated by YhcSR, we performed gel-shift assays. DNA fragments of the upstream DNA regions of *lacA* (*P_lac_,* 397 bp) and *opuCA* (*P_opuC_,* 312 bp) were obtained by PCR using the primers listed in Table 2. The amplified DNA fragments were purified and labeled with Digoxigenin using the DIG GEL Shift Kits (Roche) according to the manufacturer’s protocol. The DNA-binding and electrophoresis were performed as described [28], [29]. Briefly, the purified PCR products were labeled with Digoxigenin using terminal transferase (Roche). The labeled DNA fragments were further purified to remove the redundant DIG-ddUTP and salts. The interaction of YhcR-His with DNA was conducted in a 20 µl reaction mixture containing 0.2 pmol DIG-labeled DNA, 1 mg of poly-(dI–dC), 25 mM NaH_2_PO_4_ (PH 8.0), 50 mM NaCl, 2 mM MgCl_2,_ 1 mM DTT, 10% glycerol, and different concentrations of YhcR-His protein (final concentrations of YhcR-His were 1, 2, 4, and 6 µM corresponding to 0.5, 1, 2, and 3 µg, respectively). Unlabeled DNA fragments of the promoter region as a specific competitor was added into the reaction with 100-fold excess to labeled probe. Other unrelated proteins including BSA and SaeR (another response regulator of *S. aureus*) were used as nonspecific binding controls. The DNA binding reaction was initiated by the addition of YhcR-His and incubated at room temperature for 25 min. Samples were then loaded directly onto a 5% native polyacrylamide gel [acrylamide:bisacrylamide (29∶1) in 0.5x TBE buffer]. Electrophoresis was run for 2 h at 4°C with 7 V/cm, and the gels were transferred to Nylon membrane via electro-blotting in 0.5x TBE at 300 mA for 90 min at 4°C. After cross-linking of DNA fragments using UV, the membrane was hybridized with anti-digoxigenin-AP antibody and exposed to X-ray film for 4 hours to achieve the desired signal.

### Construction and Characterization of *Pspac*-driven Complementary Strains

In order to determine whether the modulation of *lac* by YhcSR is involved in the essentiality of the YhcSR, we created *Pspac*-driven *lacABC* operon complement strains respectively within the *yhcS* antisense expression plasmid. Briefly, The 1905 bp PCR fragment of *lacABC* (sa1995–1997) was amplified using the primers Sa1997RBSfor/Sa1995rev digested with *Not*I, then cloned into the same site of pMY107 as previously described [22]. The resulting recombinant plasmid carrying the *lacABC* genes located downstream of the *Pspac* promoter region was obtained, confirmed by PCR and DNA sequencing, and correspondingly designated as pMY307. The pMY307 recombinant plasmid was electroporated into WCUH29 and resulted in *Pspac*-driven *lacABC* genes complement strains denoted as YJ307. To examine whether the *Pspac*-driven *lacABC* genes can complement the inhibitory growth effect of *yhcS* antisense RNA, we titrated the effect of induced *yhcS* antisense RNA on growth by kinetically monitoring the growth of the above complementary strains in TSB containing Erm (5 µg/ml) and different concentrations of inducer, anhydrotertacycline (ATc) in a 96-well format using a SpectrMax plus Spectrophotometer (Molecular Devices) as previous described [10], [22]. This experiment was repeated at least three times.

## Results

### Identification of Genes Regulated by the Essential *yhcSR* System

Using a *Pspac*-regulated *yhcSR* mutant and a TetR-regulated *yhcS* antisense RNA mutant, we have demonstrated that the down-regulation of *yhcSR* expression causes a lethal effect on bacterial growth [10]. In order to elucidate the biological functions of the essential *yhcSR* system in *S. aureus*, we comprehensively examined the effect of conditional knockdown of *yhcSR* on gene expression using regulated antisense RNA technology with the combination of microarray analysis. Our preliminary microarray data showed that the down-regulation of *yhcS* expression differentially affected expression of various genes, including the *lac* operon encoding the structural genes for lactose and galactose metabolism (decreased expression two to five-fold) and *opuC* encoding a glycine betaine/carnitine/choline ABC transporter (decrease expression four to five-fold), and virulence factors (Table 3). To confirm the preliminary results, we first selected four down-regulated genes and five up-regulated genes including several essential genes and conducted qPCR analysis. For the control strain, YJ2002 (WCUH29 carrying pYH3, [30], the addition of inducer (250 ng/ml of ATc) had no significant influence on the expression of the above selected 10 genes in both microarray and qPCR assays (data not shown). However, for the *yhcS* antisense RNA expression strain JAS909, the qPCR analysis showed that the down-regulation of *yhcSR* led to 4-fold decrease of both *lacA* and *opuCA* expressions, which are consistent with the microarray data (Table 3). Interestingly, we found that the down-regulated *yhcSR* expression significantly increased the transcription of several virulence factors, including coagulase, fibronectin binding protein, and exotoxin, suggesting that the YhcSR system may function as a repressor of these virulence factors, possibly through regulation of *agr* and *saeR* (Table 3) [11].

**Table 3:** Real-time RT-PCR (qPCR) and microarray analysis of gene expression in mid-log phase of growth, using the *yhcS* antisense RNA strain.

gene	description	fold change	
		qPCR* (control)	microarray
*coa*	taphylocoagulase	+4 (0)	+5.59
*set15*	exotoxin 15	+4 (0)	+2.5
SA0959	GTP-binding elongation factor homolog	+2 (0)	+2.3
*pyrC*	dihydroorotase	+2 (0)	+2.8
*fnbB*	fibronectin-binding protein homolog	+2 (0)	+7.29
SA1269	Blt-like protein	−4 (0)	−33.4
SA1271	threonine dehydratase	−8 (0)	−51.67
*lacA*	galactose-6-phosphate isomerase	−4 (0)	−4.89
*opuCA*	glycine betaine/carnitine/choline ABC transporter	−4 (0)	−4.39

qPCR* (control): The number inside parentheses is the fold change of specific genes in control strain YJ2002 with versus without ATc. –represents down-regulated gene expression during inactivating *yhcSR* system; +represents up-regulated gene expression during inactivating *yhcSR* system.

### Confirmation of Transcriptional Regulation of *lac* using a Promoter-*lux* Reporter Fusion System

**Figure 1:**
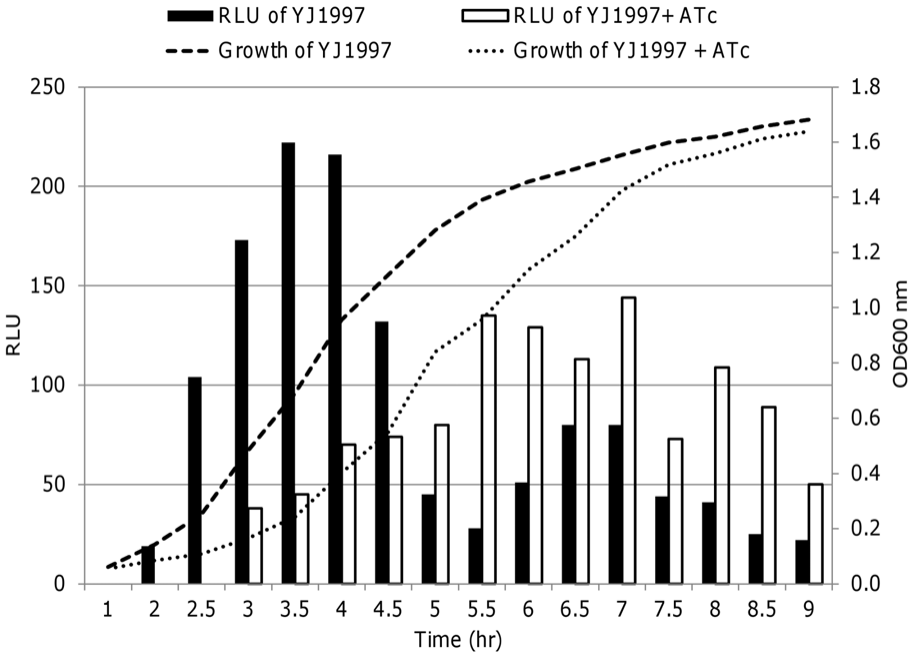
Analysis of transcriptional regulation of *lac* operon by the YhcSR system using promoter-*lux* reporter fusion. The impact of the down-regulation of *yhcSR* on the expression of *lac* operon was determined by monitoring the bioluminescence intensity during growth. The overnight cultures of *S. aureus* strains were diluted to ∼10^4^ CFU/ml with TSB containing appropriate antibiotics and in the absence (solid dots ) or presence of 200 ng/ml of inducer, ATc (solid dashes). Both bioluminescence signals and cell growth were monitored at 37°C by measuring the light intensity with a Chiron luminometer and optical density at 600 nm (OD_600_) with a SpectraMax plus Spectrophotometer every 30 min. To eliminate the effect of bacterial growth, the relative light units (RLU) were calculated (light intensity/OD_600_) from triplicate readings at different times during growth.

To further confirm whether the *yhcSR* regulator transcriptionally regulates the expression of the *lac* genes, we created a promoter-*lux* reporter fusion system using the TetR-regulated *yhcS* antisense expression vector, pCY606. The promoter-*lux* reporter fusion strains (YJ1997) and the parental control strain (YJ606) were grown in TSB in the presence of the *yhcS* antisense RNA inducer ATc (200 ng/ml) and in the absence of ATc, at 37°C with shaking. Bioluminescence intensity and optical density of the cultures were measured at different times of growth. No light signal was detected for the control strain YJ606 with and without inducer, suggesting no detectable leaky *luxABCDE* transcription (data not shown) and expression. However, for the *lac* promoter-*lux* fusion strains (YJ1997), the light intensity obviously decreased in the early log-phase of culture, but increased after later log-phase of growth during the induction of *yhcS* antisense RNA expression with inducer ATc (Figure 1). These results and in conjunction with our previous results on *opuC* [22] indicate that the *yhcSR* system transcriptionally regulates the expression of *lac* operon and *opuC* operons.

### YhcR Directly Interacts with the Upstream Regions of *lacA* and *opuCA*

To examine whether YhcR directly or indirectly regulates the expression of these essential genes, we conducted gel shift assays with the upstream regions of *lacA* and *opuCA*. Gel shift promoter probes containing the 300 to 400 bp upstream of the translational start site of the first structural gene of each operon were obtained by PCR and labeled with digoxigenin. Each gel shift assay consisted of a DNA probe-only control, the probe incubated with different concentrations of YhcR ranging from 1 to 6 µM, the probe plus 100-fold excess of unlabeled probe, and the probe with nonspecific protein BSA (2.3 µM) or another response regulator SaeR (5.6 µM).

**Figure 2:**
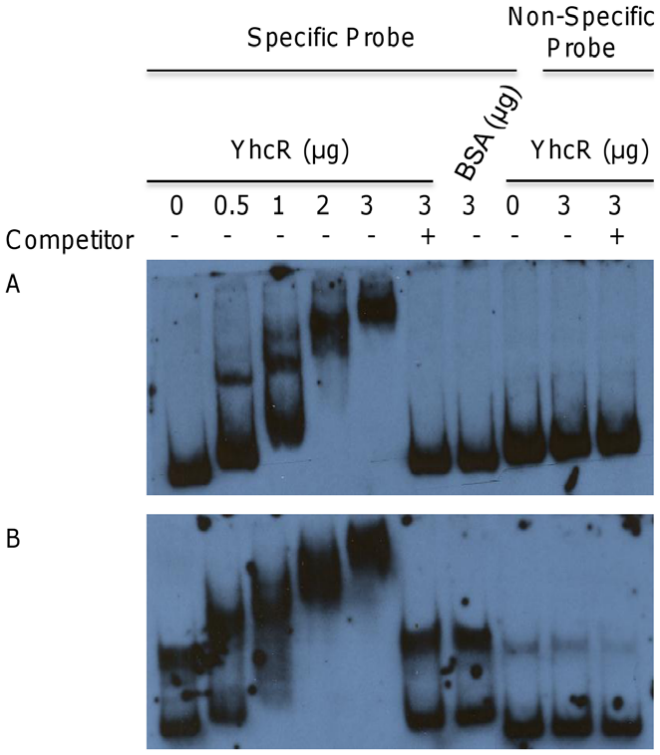
Gel shift mobility analysis of genes regulated by YhcR. Promoter regions for each gene were obtained as described. The mobility of labeled promoter fragments without addition of YhcR is shown in lane 1 (0). Different amounts of YhcR (0.5, 1, 2, 3 µg) were incubated with each DIG-labeled promoter probes: *P_lac_* (A) and *P_opuC_* (B) in 20 µl reaction volume. –represents without unlabeled specific competitor;+represents in the presence of 100-fold extra unlabeled specific competitor. Both BSA (bovine serum albumin) and SaeR (an unrelated response regulator of *S. aureus*) were used as nonspecific binding controls. Approximate 0.2 pmol of DIG-labeled promoter DNA fragment was used in each reaction.

The gel shift assays with the upstream region of *lacA* resulted in obviously shifted bands in a dose-dependent manner compared to the probe only and nonspecific proteins. Moreover, the addition of extra-unlabeled competitor apparently competed with the shifted band (Figure 2A). Furthermore, two apparent shifted bands were revealed in the gel shift analysis with the upstream region of *opuCA* probe, suggesting the possible presence of multiple YhcR binding sites in the promoter sequence. The extra-unlabeled competitor successfully competed with the shifted bands (Figure 2B). These results indicate that the YhcR response regulator may directly interact with the upstream regions of *lacA* and *opuCA* genes.

### Expression of *lacABC* Genes *in trans* Partially Complements the Lethal Effect of Down-regulating *yhcSR* Expression

**Figure 3:**
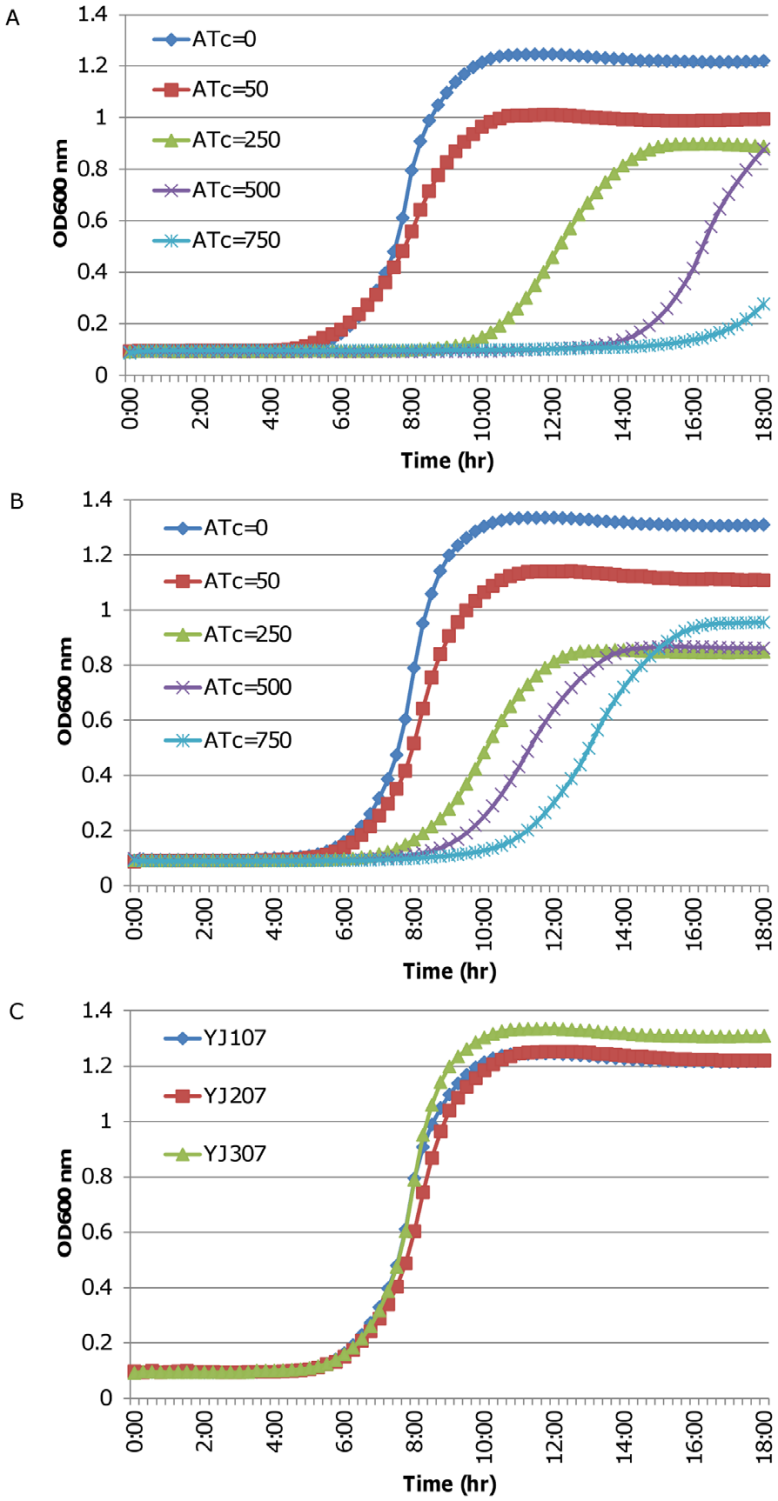
Complementation of induced *yhcS* antisense RNA by P*spac*-driven *lacABC.* Growth curves of control strain, YJ107 (A), *lacABC* complemented strain, YJ307 (B), in TSB containing 5 µg/ml of erythromycin and various concentrations of ATc (in nanograms/milliliter). (C) Represents the growth curves of the above strains in the absence of ATc. The overnight cultures of *S. aureus* strains were diluted to ∼10^4^ CFU/ml with TSB containing appropriate antibiotics and different concentrations of an inducer [anhydrotetracycline, (ATc), at concentrations of 0, 50, 250, 500, or 750 ng/ml]. Cell growth was monitored at 37°C by measuring the optical density at 600 nm (OD_600_) every 15 min, with 1 min of mixing before each reading in a SpectraMax plus Spectrophotometer. The growth curves represent one of three repeated experiments.

In order to determine whether the direct modulation of *lac* operon is associated with the essentiality of *yhcSR* system, we conducted complementary experiments using a multicopy plasmid. The *lacABC* genes were obtained by PCR using a high fidelity *pfx* DNA polymerase, and cloned downstream of *Pspac* promoter within the *yhcS* antisense RNA expression vector, pMY107 and labeled at pMY307. The recombinant plasmid was electroporated into the laboratory strain, RN4220, and subsequently, into the clinical isolate, WCUH29, labeled YJ307. The growth of the complementary strain YJ307 and control carrying parental plasmid DNA (YJ107) was kinetically monitored while increasing concentrations of inducer, ATc. Consistent with our previous findings [10], the induction of *yhcS* antisense expression with the inducer ATc dramatically inhibited the growth of the control strain (YJ107) in a dose-dependent manner (Figure 3A). In contrast, the expression of the *lacABC* genes partially restored the growth of the *yhcS* antisense RNA expression strain and shortened the differential lag-phase of growth from five to two hours in the presence of 250, 500, and 750 ng/ml of inducer ATc (Figure 3B). We previously observed similar results of partial complementation with the *opuC* operon [22].

To exclude the possibility that the above complementation may be attributed to the over-expression of the LacABC proteins or OpuCABCD proteins in the multi-copy plasmid, we compared the growth between the control strain and each complementary strain in the absence of inducer. Without the induction of *yhcS* antisense RNA expression, the complementary strain carrying the multi-copy plasmids of *lacABC* genes and the *opuC* operon showed a similar pattern of growth as the control strain showed (Figure 3C).

To determine whether the complement effect of the *lacABC* genes and *opuC* operon on the *yhcS* antisense RNA lethal function resulted from the *Pspac*-driven *lacABC* gene and *opuC* operon over-expressions *in trans*, we performed qPCR using *lacA*, and *opuCA* specific primers (Table 2). Total RNA was isolated and purified from log-phase cultures, and cDNA was reverse transcribed. The transcriptional levels of *lacA* and *opuCA* in the complementary strains (YJ307 and YJ207) respectively increased 16- and 4-fold than those in the control strain (YJ107), suggesting the complementary effect seen in the assays was due to the *Pspac*-driven expression of the *lacABC* genes and *opuC* operon.

### Expression of o*puCABCD* Genes *in trans* Enhances Bacterial Growth in High Osmotic Medium Conditions during *yhcS* Antisense RNA Induction

**Figure 4:**
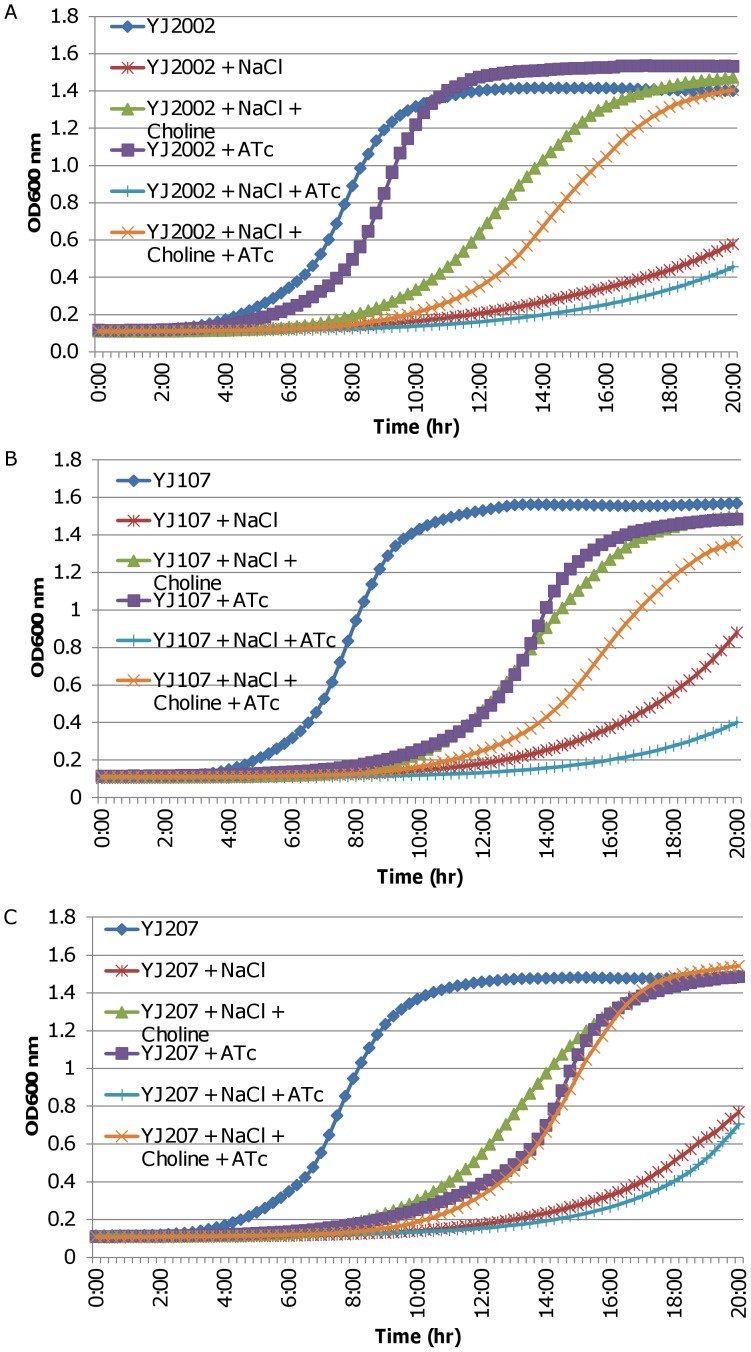
Effect of the complementation of *opuC* operon on bacterial growth in high osmotic medium conditions during the depletion of YhcSR. Growth curves of control strain, YJ2002 (A), *yhcSR* antisense RNA strain, YJ107 (B), and *opuCABCD* complemented strain, YJ207 (C) in CDM containing 5 µg/ml erythromycin and where indicated 750 ng/µl ATc, 1 M NaCl, and 1 µM choline chloride were added. The overnight cultures of *S. aureus* strains were diluted with CDM containing appropriate antibiotics and additives as indicated. Cell growth was monitors at 37°C by measuring the optical density at 600 nm (OD_600_) every 15 min, with 1 min of mixing before each reading in a BioTek Synergy II microplate reader. The growth curves represent one of three repeated experiments.

To further determine *yhcSR’s* regulation of the *opuC* operon, we conducted complementation experiments using an *opuC* operon expressing multicopy plasmid and chemically defined medium in the absence or presence of NaCl and the compatible solute, choline. Choline, oxidized in the cell to glycine betaine, is one of several osmoprotectants that *S. aureus* can use to shield itself from high osmolality environments [31], [32]. Consistent with previous reports regarding *S. aureus* growth in high osmotic conditions [24], [32], the addition of NaCl to the CDM dramatically alleviated the growth, but the addition of choline to the CDM improved the growth of the control strain YJ2002 (Figure 4A). The presence of inducer, ATc, had mild effect on the growth of *S. aureus* due to its toxicity, but the effect was equal across of the growth conditions for YJ2002 (Figure 4A). The *yhcS* antisense RNA strain YJ107 grew similarly to YJ2002 in the medium without ATc, whereas the induction of *yhcS* antisense RNA with ATc caused a remarkable growth defect, which was further exacerbated by adding NaCl to the CDM (Figure 4B); whereas adding choline to the NaCl-CDM partially recovered YJ107 growth (Figure 4B). The presence of the *Pspac*-driven *opuCABCD* genes in YJ207 resulted in *S. aureus* growing better in CDM containing NaCl and ATc, indicating protection of the cells from the high salt conditions (Figure 4C). Moreover, the addition of choline to the NaCl-ATc-CDM further enhanced the growth of YJ207 (Figure 4C). Taken together, the above data indicate that the *Pspac*-driven expression of *opuCABCD in trans* is able to complement the function of the down-regulated endogenous *opuCABCD* by YhcSR.

## Discussion

Two-component signal transduction systems play important roles in the ability of bacteria to adapt to various environments by sensing alterations in their surroundings and by altering gene expression [5]. In *S. aureus* genomes, at least 16 pairs of two-component signal transduction systems have been revealed [33]. However, most of them have not been functionally explored. Previous work in our laboratory led to the identification of a novel essential two-component signal transduction system, *yhcSR,* in *S. aureus* [10] and demonstrated its role in regulating disimilatory nitrate reduction [21]. In this study, we revealed that YhcSR is a global regulator and investigated two additional target genes that are directly regulated by the essential YhcSR system.

Our results demonstrate for the first time that the novel essential YhcSR system directly regulates the expression of *lac* and *opuC* operons [34], [35]. Using microarray approaches, we and other investigators have successfully identified genes that are directly and/or indirectly regulated by different regulators including two-component signal regulatory systems, such as ArlRS [26], SaeRS [36], AgrA and SarA [37], and MgrA [38]. In this study, we employed a similar approach to identify genes that are modulated by the YhcSR system. Our preliminary microarray data showed that the depletion of the YhcSR system apparently down-regulates the expression of *lac* and *opuC* operons, which were confirmed by semi-quantitative RT-PCR analysis. To further confirm whether YhcSR is able to regulate the expression of the *lac* operon at the transcriptional level, we performed promoter-*lux* reporter fusion assays. Consistent with the RT-PCR data, the down-regulation of *yhcSR* expression obviously decreased the bioluminescence intensity of the *Plac-lux* promoter fusion. Taken together, and in conjunction with our previous publication [22] results indicate that the essential *yhcSR* system transcriptionally regulates the *lac* and *opuC* operons in *S. aureus*. The different profiles of *lux* expression in the different promoter-*lux* reporter fusions during different stages of growth suggest that in the early log-phase YhcR controls *lac* transcription; in contrast, in the middle and late log-phases YhcR positively modulates *opuC* operon expression [22]. This finding is not surprising since it has been found that the well-studied *agr* system differentially regulates the expression of both cell-wall associated proteins and exported toxins in different phases of cell growth [33].

In order to elucidate whether the above regulatory effects on the *lac* and *opuC* operons expression are mediated directly by YhcR or indirectly through other regulators, we employed gel shift assays. The upstream promoter regions of the *lac* and *opuC* operons bound to YhcR-His in a dose-dependent manner. We also found that the upstream promoter regions showed multiple shifted bands with a low concentration of YhcR, suggesting that these promoter regions may bind YhcR as a dimer or at multiple sites. This phenomenon has been revealed in different regulators, including OmpR, SarA, and SrrA [29], [39], [40]. These results indicate that YhcR positively mediates the expression of the *lac* and *opuC* operons by directly binding to their promoter regions.

In addition, unlike OmpR its binding affinity to its target DNA is increased by phosphorylation [39], [41]; the phosphorylation of YhcR had no obvious impact on our gel shift assays (data not shown), which is consistent with the report of SrrA [40]. We cannot dismiss the possibility that the *in vitro* phosphorylation of YhcR in this study is not as effective as expected. It remains to be determined whether a phosphorylated YhcR has different binding sites and/or affinities in the promoter regions of *lac* and *opuC*.

To address whether the above transcriptional effects of YhcSR on *lac* expression are relevant to the biological function or essentiality of the *yhcSR* system, we conducted complement experiments by introducing *Pspac-*driven *lacABC* genes in a multicopy plasmid. The complementation of the *lacABC* dramatically curtailed the inhibitory effect of the induced *yhcS* antisense RNA. Biochemical evidence indicates YhcSR is an oxygen sensing two-component system [11], thus regulation of the *lac* operon by YhcSR links lactose fermentation to the absence of oxygen. The importation and ultimate fermentation of lactose provides an additional energy source during oxygen-limited growth.

A similar result of partial complementation was observed for *Pspac-*driven *opuC* operon [22]. Additionally, we found that the down-regulation of *yhcSR* expression effectively inhibited bacterial growth, whereas the complementation of the *opuC* operon enhanced bacterial growth of the bacteria in the high osmotic medium, and the supplementation of the high osmotic medium with choline restored bacterial growth to that equal of the cells in CDM with ATc alone. These data indicate that YhcSR is involved in the modulation of the transportation of the osmoprotectant choline through direct regulation of *opuC* operon expression. The transport of choline by OpuCABCD and their role in osmoprotection has been well established [24], [42]–[44]. In *S. aureus*, OpuCA interacts with and influences the level of phosphorylated TRAP protein, a protein that protects DNA from oxidative stress [24], [45]. The kinase activity of YhcS is influenced by oxidative stress (such as H_2_O_2_) [11] and the regulation of *opuC* by YhcSR is likely part of the overall stress response that occurs when *S. aureus* encounters stressful environmental conditions. The upregulation of multiple stress response pathways likely allows the cell to better handle additional environmental insults.

We have previously used *yhcS* antisense RNA to demonstrate the essential nature of YhcSR in a hospital-associated (HA)-MRSA strain, WCUH29 [10], [21]. A recent publication suggested the essential nature of YhcSR was strain specific, as their MSSA strain Newman *yhcS* antisense RNA strain had no growth defect [11]. To investigate this claim, we introduced our *yhcS* antisense RNA plasmid into *S. aureus* Newman and community associated (CA)-MRSA *S. aureus* 923 (supplementary Figure S1). Contrary to the other authors’ results, we found *S. aureus* Newman to be extremely sensitive to our *yhcS* antisense RNA. The *yhcS* antisense RNA Newman strain exhibited poorer growth without induction of the antisense RNA compared to the empty vector control; and severely inhibited growth was detected with as little as 250 ng/µl of antisense RNA inducer, anhydrotetracycline (ATc) in TSB. The other authors did not provide evidence that their antisense RNA actually reduced *yhcS* RNA or protein product, thus it is likely that their antisense RNA was ineffective and did not work as designed. We have previously demonstrated *yhcS* RNA knockdown and YhcR protein loss using our *yhcS* antisense RNA plasmid after ATc induced expression [10]. A similar growth defect was seen in *S. aureus* 923, but higher induction of the *yhcS* antisense RNA was needed to inhibit growth. The difference in sensitivity to the *yhcS* antisense RNA between MSSA Newman and the MRSA strains may be due to their genetic differences [8], [15]. Taken together, the above data indicate that the YhcSR system is essential for all *S. aureus* strains tested, including HA-MRSA, CA-MRSA, and MSSA.

Structural alignment indicates that YhcS and YhcR are more than 40% identical to YhcY and YhcZ in *B. subtilis*. However, unlike the YycFG system which is essential for *B. subtilis* and *S. aureus* [9], [12], YhcYZ is dispensable for *B. subtilis* growth [46]. The YhcSR system may play different roles in *S. aureus* compared to the YhcHZ system in *B. subtilis*. In *B. subtilis*, only limited genes are regulated by YhcHZ [47]. In contrast, in *S. aureus* our preliminary microarray results showed that more than 80 genes are modulated by YhcSR (data not shown). This is also true for the YycFG system because YycF is involved in the regulation of the FtsAZ operon in *B. subtilis* [14], whereas there is no such evidence in *S. aureus* and *S. pneumoniae* [16], [18]–[20], [33], [48].

Interestingly, our preliminary microarray and qPCR analyses also indicate that the essential YhcSR system is probably a repressor of virulence factors because the down-regulation of *yhcSR* expression dramatically increased the expression of coagulase, fibronectin binding protein, and exotoxin (Table 3), possibly through regulation of the *agr* and *sae* operons [11]. This suggests that the YhcSR system may function as a global regulator.

## Supporting Information

Figure S1 **Growth of** ***S. aureus*** **Newman and** ***S. aureus*** **923 with** ***yhcS*** **antisense RNA plasmid.** MSSA *S. aureus* Newman and MRSA *S. aureus* 923 were electroporated with the control plasmid, pYH3, or the *yhcS* antisense RNA plasmid, pSAS909 [10]. Overnight cultures of *S. aureus* strains were diluted to ∼10^4^ CFU/ml with TSB containing 5 µg/ml of erythromycin and different concentrations of an inducer [anhydrotetracycline, (ATc), at concentrations of 0, 100, 250 ng/ml]. (A) Represents the growth curves of the control Newman strain and *yhcS* antisense RNA strain, Newman-606 and (B) represents the MRSA923 control and *yhcS* antisense RNA strain, MRSA923-606. Cell growth was monitored at 37°C by measuring the optical density at 600 nm (OD_600_) every 15 min, with 1 min of mixing before each reading in a BioTek Synergy II Microplate Reader. The grow curves represent one of three repeated experiments.
(TIFF)
