# Supplementary figures and images for: The Essential yhcSR Two-Component Signal Transduction System Directly Regulates the lac and opuCABCD Operons of Staphylococcus aureus
Source: PLoS One. 2012 Nov 30;7(11):e50608. doi: 10.1371/journal.pone.0050608 (PMC3511567; doi:10.1371/journal.pone.0050608)

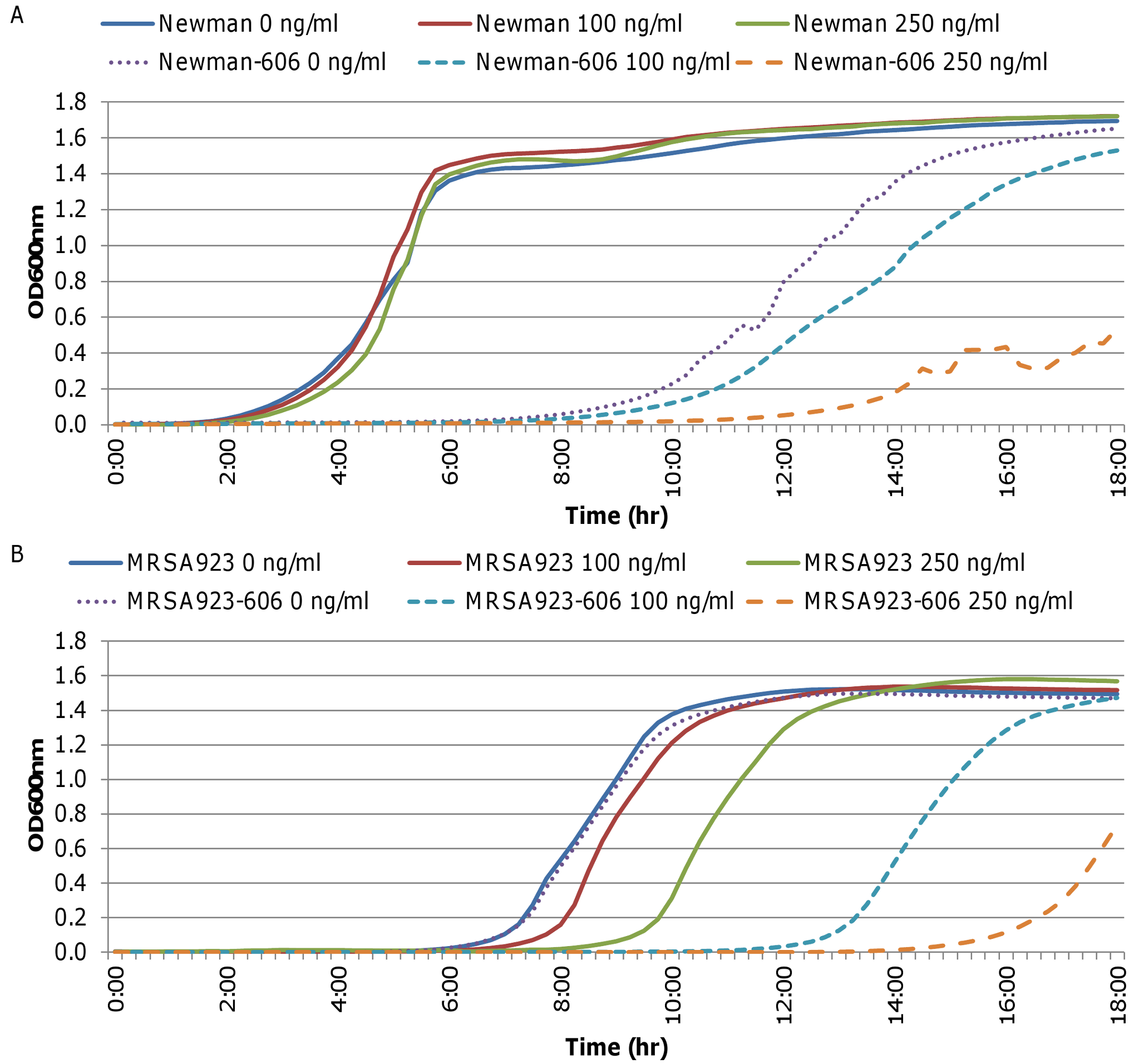

Supplement: Figure S1 — Growth of S. aureus Newman and S. aureus 923 with yhcS antisense RNA plasmid. MSSA S. aureus Newman and MRSA S. aureus 923 were electroporated with the control plasmid, pYH3, or the yhcS antisense RNA plasmid, pSAS909 [10]. Overnight cultures of S. aureus strains were diluted to ∼104 CFU/ml with TSB containing 5 µg/ml of erythromycin and different concentrations of an inducer [anhydrotetracycline, (ATc), at concentrations of 0, 100, 250 ng/ml]. (A) Represents the growth curves of the control Newman strain and yhcS antisense RNA strain, Newman-606 and (B) represents the MRSA923 control and yhcS antisense RNA strain, MRSA923-606. Cell growth was monitored at 37°C by measuring the optical density at 600 nm (OD600) every 15 min, with 1 min of mixing before each reading in a BioTek Synergy II Microplate Reader. The grow curves represent one of three repeated experiments. (TIFF) [file pone.0050608.s001.tiff]
